# Supplementary material for: A Silicon Sub-Bandgap Near-Infrared Photodetector with High Detectivity Based on Textured Si/Au Nanoparticle Schottky Junctions Covered with Graphene Film
Source: Sensors (Basel). 2023 Jul 6;23(13):6184. doi: 10.3390/s23136184 (PMC10346632; doi:10.3390/s23136184)
Supplement: Supplementary file 1 [file sensors-23-06184-s001.zip › sensors-2431873-supplementary.pdf]

Supplementary Information

A Silicon Sub-bandgap Near-Infrared Photodetector with High Detectivity Based on Textured Si/Au Nanoparticle Schottky Junctions Covered with Graphene Film

Xiyuan Dai [1], Li Wu [1], Kaixin Liu [1], Fengyang Ma [1], Yanru Yang [1], Liang Yu [1], Jian Sun [1,2,*] and Ming Lu [1,2,*]

[1] Department of Optical Science and Engineering, and Shanghai Ultra-Precision Optical Manufacturing Engineering Center, Fudan University, Shanghai 200433, China; 20110720001@fudan.edu.cn (X.D.); 21110720018@m.fudan.edu.cn (L.W.); 21210720009@m.fudan.edu.cn (K.L.); 22110720009@m.fudan.edu.cn (F.M.); 22210720005@m.fudan.edu.cn (Y.Y.); 21210720284@m.fudan.edu.cn (L.Y.)

[2] Yiwu Research Institute, Fudan University, Yiwu 322000, China

* Correspondence: jsun@fudan.edu.cn (J.S.); minglu55@fudan.edu.cn (M.L.)

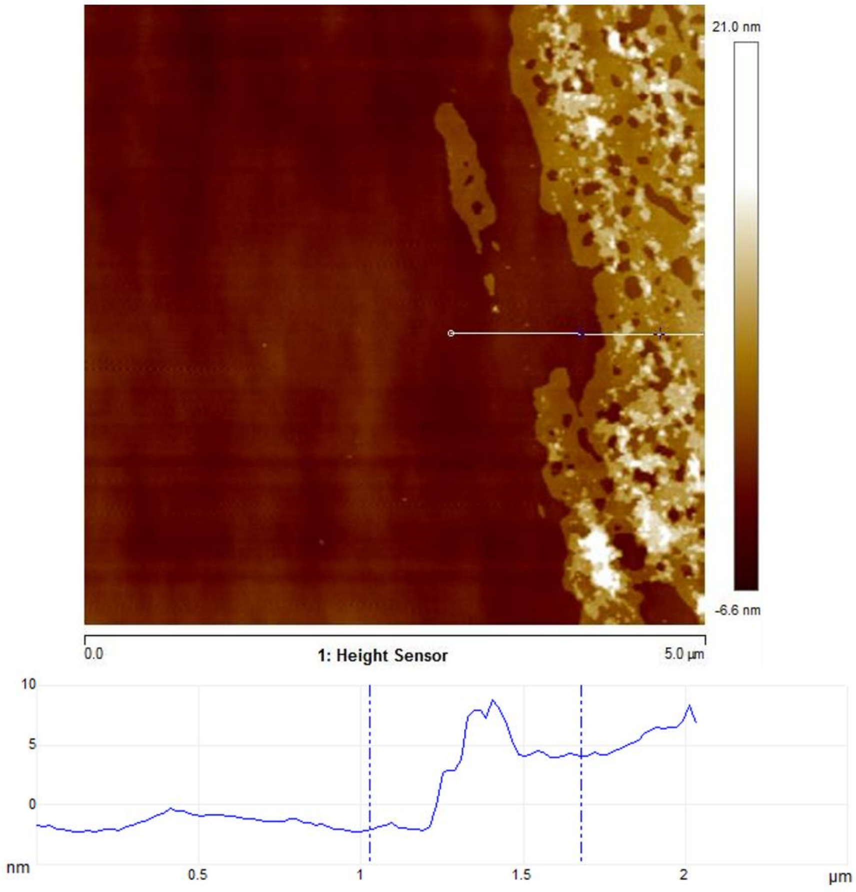

Figure S1. AFM image of the graphene film spin-coated on the planar Si. A step was made to obtain the thickness of graphene film.

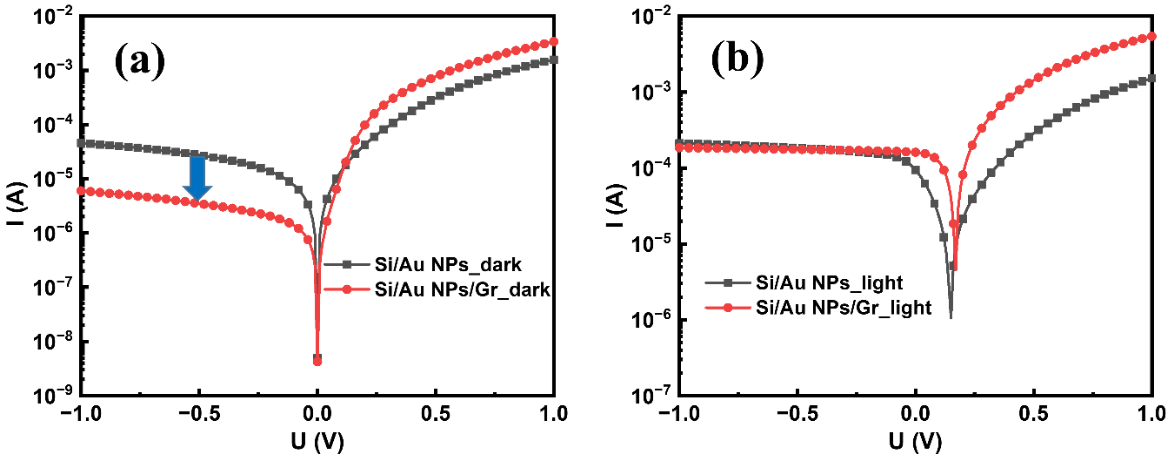

Figure S2. I-V characteristics of planar Si/Au NPs and planar Si/Au NPs/Gr PD under dark condition (a) and 1319 nm light illumination with incident power of 0.1 W (b).

To verify the role of Gr film, PDs based on the planar Si were fabricated. The dark current under reverse bias was reduced and responsivity at 0 V increased from 0.9 mA/W to 1.6 mA/W after introducing Gr film. These results show the reproducibility of Gr-induced better performance.

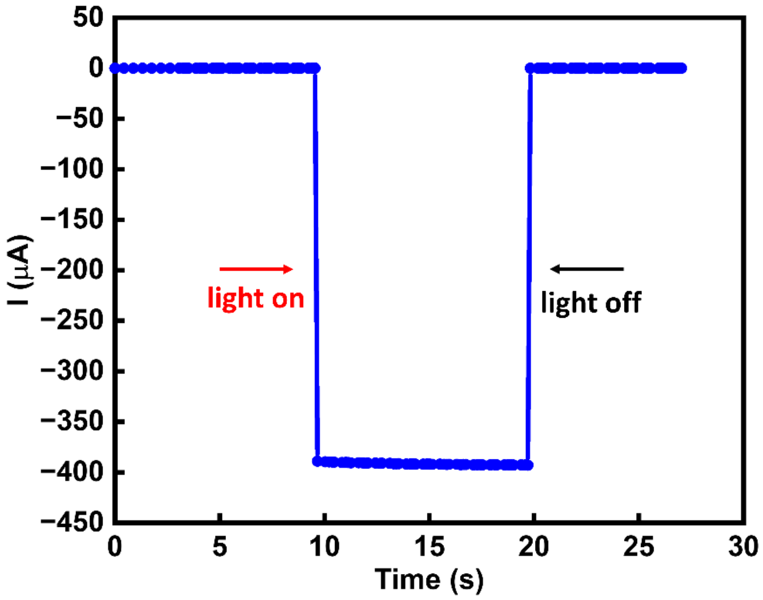

Figure S3. Photoresponse of Si/Au NPs/Gr PD device working at zero bias and -60 ℃ under 0.1 W 1319 nm light illumination.
